# Long-Term Apparent Temperature, Extreme Temperature Exposure, and Depressive Symptoms: A Longitudinal Study in China

**DOI:** 10.3390/ijerph20043229

**Published:** 2023-02-12

**Authors:** Jianbo Jin, Zhihu Xu, Ru Cao, Yuxin Wang, Qiang Zeng, Xiaochuan Pan, Jing Huang, Guoxing Li

**Affiliations:** 1 Department of Occupational and Environmental Health Sciences, Peking University School of Public Health, Beijing 100191, China; 2 Department of Occupational Disease Control and Prevention, Tianjin Centers for Disease Control and Prevention, Tianjin 300011, China

**Keywords:** depressive symptoms, apparent temperature, extreme temperature, longitudinal study

## Abstract

Temperature is increasingly understood to impact mental health. However, evidence of the long-term effect of temperature exposure on the risk of depressive symptoms is still scarce. Based on the China Health and Retirement Longitudinal Study (CHARLS), this study estimated associations between long-term apparent temperature, extreme temperature, and depressive symptoms in middle-aged and older adults. Results showed that a 1 °C increase or decrease from optimum apparent temperature (12.72 °C) was associated with a 2.7% (95% CI: 1.3%, 4.1%) and 2.3% (95% CI: 1.1%, 3.5%) increased risk of depressive symptoms, respectively. This study also found that each percent increase in annual change in ice days, cool nights, cool days, cold spell durations, and tropical nights was associated with higher risk of depressive symptoms, with HRs (95%CI) of 1.289 (1.114–1.491), 2.064 (1.507–2.825), 1.315 (1.061–1.631), 1.645 (1.306–2.072), and 1.344 (1.127–1.602), respectively. The results also indicated that people living in northern China have attenuated risk of low apparent temperature. Older people were also observed at higher risk relating to more cool nights. Middle-aged people, rural residents, and people with lower household income might have higher related risk of depressive symptoms due to increased tropical nights. Given the dual effect of climate change and global aging, these findings have great significance for policy making and adaptive strategies for long-term temperature and extreme temperature exposure.

## 1. Introduction

It is generally agreed that a range of factors contribute to the development of depression, including biological, environmental, genetic, and psychological factors [1,2]. One environmental factor that has been reported to have an impact on the incidence of depression is temperature, with both high or cold temperatures having been shown to have acute effects on the occurrence of depression, such as increased admissions to emergency departments and hospitalizations [3,4,5,6,7]. However, the long-term effects of temperature on depression have been found inconsistent in previous studies. While a study in Ireland found no significant association between annual average temperature and the risk of depression [8], studies in Spain and Taiwan have suggested that higher annual average temperature may be related to an increased risk of depression [9,10]. In addition, given the potential impacts of climate change, it is important to investigate the effects of increasing extreme temperature events on mental health [11].

Several limitations have plagued research on the relationship between long-term temperature and depression. First, previous studies have often been conducted in regions with a limited range of climate conditions [9], and few have explored the effects of low temperature in cold climates. Second, few studies have considered the influence of various meteorological factors on mental health outcomes. While ambient temperature is a key factor in human thermal perception, other factors such as relative humidity and wind speed can also play a role. The concept of apparent temperature, which combines air temperature and other meteorological factors, may provide a more comprehensive assessment of thermal feeling [12], and more and more studies focused on the thermal environment [13,14]. Although previous studies have found a relationship between apparent temperature and mental health outcomes [15,16,17], the impact of apparent temperature on depression has not been extensively investigated. 

In this study, we first explored the long-term effect of temperature on depressive symptoms for middle-aged and older adults based on the China Health and Retirement Longitudinal Study (CHARLS) [18], which repeatedly measured the depressive disorder of a nationally representative sample of middle-aged and older Chinese adults in 2011, 2013, 2015, and 2018 [19]. We also investigated the impact of the annual change in extreme temperature events on depressive symptoms.

## 2. Materials and Methods

### 2.1. Data Source and Processing

We collected data from the China Health and Retirement Longitudinal Study (CHARLS), which is a national representative longitudinal survey of Chinese adults over the age of 45 (https://charls.charlsdata.com, accessed on 15 March 2021). Using the multi-stage probability sampling method, CHARLS selected 150 county-level units from 450 communities in 125 cities of 28 provinces of China [20]. The survey includes four waves, including the 2011–2012 baseline (W1) survey, with 17 596 individual participants recruited; the second wave (W2) survey, with 15,179 followed in 2013–2014; the third wave (W3) survey, with 13,002 followed in 2015–2016; and the fourth wave (W4) survey, with 11,486 followed in 2017–2018. 

We excluded 4952 participants with depressive symptoms and 6878 participants who did not provide depressive symptom information at W1, W2, W3, or W4 or who withdrew from the survey to identify a group of respondents without depressive symptoms at baseline. We excluded 158 participants for lack of covariate information and 8 participants in cities with few cases (Lanzhou and Shenzhen). Our final analysis sample included 5600 individuals who had no depressive symptoms at baseline, and then provided complete data for all study variables in W1, W2, W3, and W4 (Figure S1). The likelihood of non-responses seemed to not correlate with specific characteristics (4555 participants without information of depressive symptoms or in withdrawal group) [20]. 

CHARLS obtained ethical approved for collecting data on human subjects by Peking University Institutional Review Board and all participants provided written informed consent.

### 2.2. Variable Design 

#### 2.2.1. Depressive Symptom Measurement

We used a binary variable of having depressive symptoms or not. The 10-item short form of the Center for Epidemiologic Studies Depression Scale (CESD-10) was used to measure depressive symptoms. CESD-10 has been commonly used to examine depressive symptoms among Chinese middle-aged and older adults with satisfactory validity and reliability [21]. Scores in the range of 0–30 indicated the degree of depressive symptoms. The cut-off point of 10 was used to generate the binary depressive symptoms variable (1 = yes, 0 = no) [22]. Those who had scores above 10 were classified as having depressive symptoms. Details of the CESD-10 are described in the Appendix.

#### 2.2.2. Temperature Exposure Assessment

In this study, we adopted apparent temperature as the temperature exposure. Apparent temperature, as a comprehensive index of ambient temperature, relative humidity, and wind speed, is more objective to reflect human body perception than the average ambient temperature [12,23]. The daily meteorological information (ambient temperature, relative humidity, wind speed, and sunlight hours) of all selected cities in the same period (2011–2018) was obtained from the China Meteorological Science Data Sharing Service Network: (http://data.cma.cn/data/cdcdetail/dataCode/SURF_CLI_CHN_MUL_DAY.html, accessed on 15 March 2021). We first calculated daily apparent temperature by using the original data of each weather station, and further calculated the monthly averages. The apparent temperature was calculated by the following equation [17]:

(1) AT=Ta+0.33∗e−0.70∗WS−4.00

(2) e=Rh/100*6.105∗exp(17.27∗Ta/(237.7+Ta))

where *Ta* is the temperature (°C), *Rh* is the relative humidity (%), and *WS* is the average wind speed (m/s). 

Nearest-neighbor interpolation was applied to estimate the monthly data across the mainland of China at a spatial resolution of a regular grid of 0.1° × 0.1° (10 km × 10 km). Due to confidential reasons, the subjects of CHARLS could only be geocoded to city-level codes. Therefore, we matched the longitude and latitude of the city centroid with the regular grids. We utilized the internal average temperature between two waves of survey as the exposure values.

The tenfold cross validation (CV) method was used to validate the accuracy of estimated temperature. The results of tenfold CV are shown in Figure S2, and the estimates were closely compliant with monthly in situ observations (R^2^ = 0.95; RMSE = 2.34 °C).

We constructed extreme temperature indices considering previous studies [24,25], using the daily maximum and minimum ambient temperatures in the period of 2010–2018 for each city. We finally chose ten typical extreme indices, as shown in Supplementary Table S1. The annual changes in extreme temperature indices in the period of 2011–2018 were calculated by the following equation:

(3) $$annual\;change=\frac{extreme\;temperature\;indice_{i}-extreme\;temperature\;indice_{i-1}}{extextreme\;temperature\;indice_{i-1}}$$

where *i* indicates the year from 2011–2018. 

#### 2.2.3. Covariates

Recorded demographic characteristics (age, sex, and marriage) and socioeconomic status covariates (education attainment, urbanicity of residence, and household income per capita) were extracted from CHARLS. The city-level per capita gross domestic product (GDP) was also collected from the statistical yearbook, the National Bureau of Statistics, and China’s national knowledge infrastructure. Age was divided into middle-age and old by cut-off of 65 years. Household income status was divided into binary by average income. Educational attainment was divided into binary of whether participants attained junior school. By the Kunlun–Qinling–Huaihe River line, the cities were divided into northern and southern cities, with or without heating policy. The index table of variates is shown in Supplementary Table S2.

### 2.3. Statistical Analyses

We assessed the association between temperature and the risk of depressive symptoms using a time-varying Cox proportional hazards model on a year-based time scale. The proportional hazards assumption was tested using Schoenfeld residuals. The visualization of the conceptual causal pathway is shown as a directed acyclic graph (DAG) in Supplementary Figure S3. We adjusted for age (continuous), sex (male vs. female), marriage (married or single), education attainment (higher than primary school vs. primary school or lower), urbanicity of residence (urban vs. rural), household income status (higher than average or lower than average), annual average sunlight hours (log), and city-level GDP (continuous).

Firstly, we used punitive spline regression (df = 3) to analyze the exposure–response curve of long-term exposure to temperature and depressive symptoms. After nonparametric estimation, optimum temperature with lowest risk would be the cut-off to stratify temperatures into high and low temperatures. Secondly, we estimated the association between annual change in extreme temperature and depressive symptoms, additionally controlling for annual average apparent temperature (log) and annual average sunlight hours (log). 

We conducted several sensitivity analyses to confirm the robustness of our results. First, we repeated the main analyses by using over 12 scores of CEDS-10 as the cut-off. Second, due to different duration times in 3 years between 2015–2018, we repeated the main analyses by using the moving average apparent temperature of the 2 years before each survey as the temperature exposure. Third, we used annual average ambient temperature as exposure to explore the exposure–response curve.

We evaluated the modification in the association between long-term temperature and depressive symptoms by stratifying age (≥65 years and <65 years), sex (male or female), urbanicity of residence (rural or urban), household income status (higher than average or lower than average), education attainment (primary school or below, or junior high school or above), and geographic location (living in northern or southern cities). Effect modification analysis was performed by adding an interaction term between temperature and the testing variable in the model.

Data arrangement, cleaning, and all statistical analyses were conducted using R (version 4.0.2) with packages dplyr, survival, and smoothHR. Statistical significance was defined as *p* < 0.05, two sides.

## 3. Results

### 3.1. Descriptive Statistics

**Figure 1:**
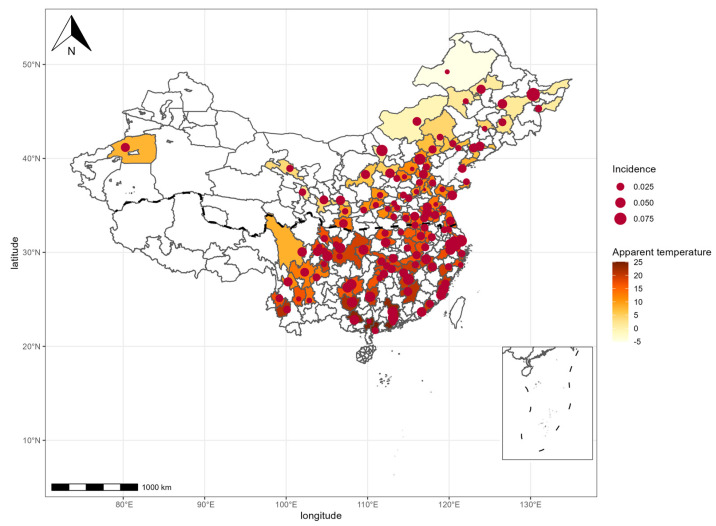
Map of average annual apparent temperature and incidence of depressive symptoms in each city (2011−2018). Dashed line refers to Kunlun−Qinling−Huaihe River line, dividing cities into the south and the north. Small islands or areas with missing data are not displayed in that map.

We finally included 5600 participants without depressive symptoms at baseline (mean age of 57.18 yr; 52.3% male; 92.7% married; 64.6% with education higher than primary school; 39.7% in urban areas) (Table 1 and Supplementary Table S3). In cities of CHARLS, the average annual apparent temperature between 2011 and 2018 ranged from −6.89–25.94 °C (median: 14.94 °C). During the follow-up period, 2457 of participants were followed with depressive symptoms. Participants were detected to have depressive symptoms in 5.61 years on average. Average annual apparent temperature and incidence rate of depressive symptoms in 2011–2018 are shown in Figure 1. It was obvious that apparent temperature was significantly higher in the south than in the north. The incidences of depressive symptoms in each city in China were between 0.71% and 9.09%, and the mean annual incidence was 2.85%.

### 3.2. Main Results

**Figure 2:**
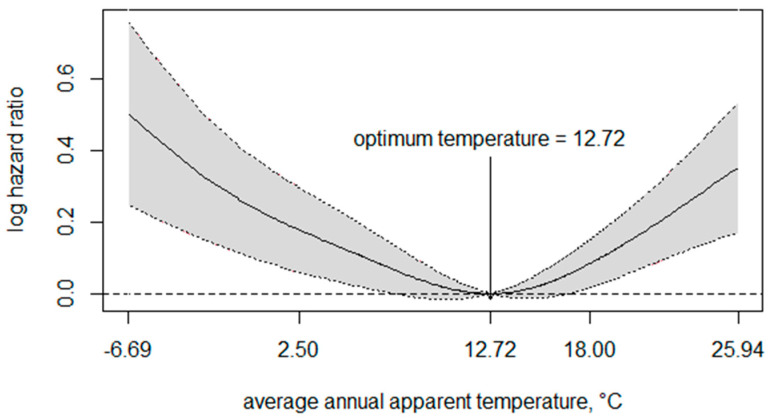
The exposure−response curve of long-term temperature exposure and depressive symptoms. Age, sex, marriage status, household income, education attainment, urbanicity of residence, gross domestic product (GDP), and annual average sunlight hours were adjusted. The solid line represents log hazard ratio, and gray zone indicates 95% confidence interval.

After adjusting for covariates such as age, sex, marriage status, household income, education attainment, urbanicity of residence, city-level GDP, and annual average sunlight hours, the long-term effect of low/high apparent temperature on depression shows to be non-linear (Figure 2). Long-term exposure to both low and high apparent temperature tended to increase the risk of depressive symptoms.

Based on the exposure–response curve of long-term exposure to apparent temperature and depressive symptoms, we stratified average apparent temperatures into high and low temperatures. The optimum apparent temperature (12.72 °C) was chosen as the cut-off. High or low apparent temperature referred to apparent temperature higher or lower than 12.72 °C, respectively. Table 2 showed the risks of high and low apparent temperature, in that the risk of depressive symptoms increased by 3.2% and 2.4% when the apparent temperature was higher or lower than 12.72 °C, respectively (Table 2). 

We also observed that each percent increase of annual change in ice days, cool nights, cool days, and cold spell durations was associated with higher risk of depressive symptoms, with HRs (95%CI) of 1.289 (1.114–1.491), 2.064 (1.507–2.825), 1.315 (1.061–1.631), and 1.645 (1.306–2.072), respectively. For the annual change in extreme hot temperatures, the change in tropical nights was associated with depressive symptoms, with an HR (95%CI) of 1.344 (1.127–1.602).

### 3.3. Modification

**Figure 3:**
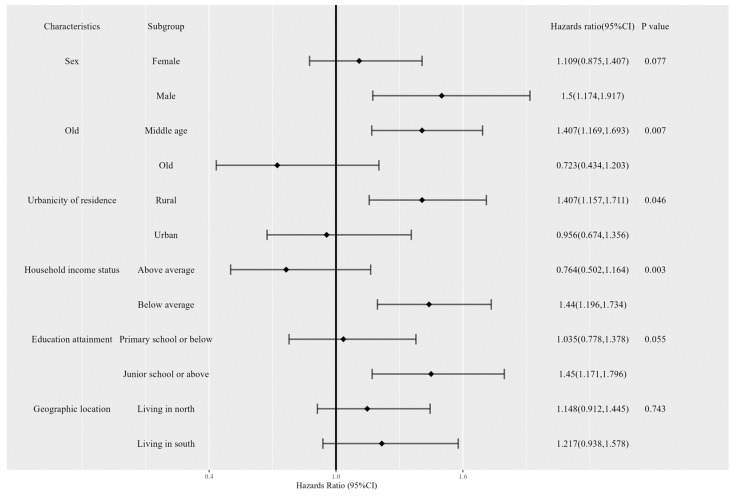
Hazards ratios of depressive symptoms, each percent increase in annual change in tropical nights by baseline characteristics.

We estimated the effect of long-term temperature and extreme temperature in different subgroups and checked the significance of modification. For participants exposed to low apparent temperature, a significant difference was found in the long-term low-apparent-temperature-related risk between participants living in northern (hazard ratio and 95%CI: 1.031 (1.018,1.044)) and southern cities (hazard ratio and 95%CI: 1.115 (1.053,1.181)) (interaction *p* value = 0.014) (Table 3). In addition, older people seem to have higher risk relating to more cool nights (interaction *p* value = 0.007) (Supplementary Figure S5). As for more tropical nights, middle-aged people, rural residents, and people with lower household income might have higher related risk of depressive symptoms (interaction *p* values: 0.007, 0.046, and 0.003, respectively) (Figure 3). No other significance was observed, and the results of stratification analysis are shown in Supplementary Figures S4–S7.

### 3.4. Sensitivity Analyses

To confirm the robustness of our results, we conducted several sensitivity analyses. The main results are still robust, as shown in Supplementary Table S4. We chose a score of 12 as the cut-off point to classify depressive symptoms and used the moving average apparent temperature of the year before each survey as the temperature exposure. We also found the robust U-shaped exposure–response curve when using annual ambient temperature as exposure (Supplementary Figure S8).

## 4. Discussion

This study found a U-shaped exposure response curve between depressive symptoms and long-term temperature exposure, and both high and low apparent temperatures increased the risk of depressive symptom incidence. Extreme hot and cold were significantly associated with depressive symptoms. People living in southern China might be more sensitive to low temperature, and older people perhaps suffered more depressive symptom risk confronting more extreme cold events. Middle-aged people, rural residents, and people with low household income might have higher extreme-heat-related risk of depressive symptoms.

A study conducted in Taiwan also found a non-linear exposure–response relationship between long-term temperature and depression, but found no significant effects of low temperature (with a threshold temperature of around 23 °C) [9]. In addition, a study in Spain found a significant association only in regions with the highest level of temperature [10]. However, there might be several reasons for this discrepancy in findings. One is the difference in the depression measurement, as the study in Taiwan measured major depressive disorder and the study in Spain measured depression only by self-reported diagnosed depression and antidepressant treatment, while our study measured depressive symptoms. Additionally, the temperature range in our study was wider than that of Taiwan and Spain (ambient temperature: −1.76–23.38 °C in our study vs. 16.5–25.4 °C in Taiwan vs. 11.4–20.2 °C in Spain). Using Figure S8, we can visually explain the inconsistency over a wider range of temperatures. Annual average ambient temperature in Spain was around optimum temperature, showing that only the highest level of temperature was associated with depression, while temperature in Taiwan was above optimum temperature, showing a significant impact of high temperature but no significant impact of low temperature.

### 4.1. Explanation of the Potential Mechanism, and Modification

The observed association may be plausible in the biological mechanism. Both heat and cold stress may affect alterations in autonomic function [26,27,28], in which prolonged imbalance may trigger the chronic low-grade inflammatory reaction, a potential cause of depression [29]. Humidity may also play a role in this relationship by affecting the body’s ability to cool down through sweat evaporation, potentially exacerbating the effects of extreme heat on mental health in vulnerable individuals [30]. Additionally, increased nighttime temperature may disturb sleep–wake cycles governed by circadian rhythms [31], which induces excessive activity of the Hypothalamic–Pituitary–Adrenal (HPA) Axis and further mental disorders [32]. Specifically, for those who are prone to acute or chronic mental problems, the long-term inability to get rid of high temperature might cause irritability and intermittent psychological distress [33]. Reduced cutaneous thermal sensitivity and subjective thermal perception during cooling may also make older people more susceptible to cold exposure [34]. However, the biological mechanism of how high or low temperature affects depressive symptoms needs further investigation. 

The results of this study showed that the effects of low temperature on depression vary between northern and southern regions in China. This may be interpreted as the result of the China’s Huai River policy, which provides central heating or low-cost indoor heating during the winter to cities north of the Huai River, but not to those in the south. In northern China, people use either central heating provided by local systems or domestic stoves burning subsidized bulk coal for heating [35]. China is facing a dilemma, in which the results in this study suggest that heating policy is associated with decreased temperature-related depressive symptoms, but both heating modes release air pollutants that damage human health [36]. Therefore, it is important to promote more efficient, clean heating strategies, especially in the construction of future heating systems in cities that are affected by low temperatures. 

Younger individuals and those with low socioeconomic status were reported to be more vulnerable to heat-related anxiety and mood disorders [37,38], which is consistent to our results shown in Figure 3. However, other research has indicated that older individuals may be the most vulnerable to extreme temperature [9,10,39]. It is necessary to conduct further studies using large datasets that include various mental disorders to determine the modifying effects and identify vulnerable populations in order to implement effective prevention strategies.

### 4.2. Strengths and Limitations

This study has several strengths. The study provided novel findings of the non-linear association between long-term apparent temperature exposure and depressive symptoms. Our findings suggested that besides extreme hot, extreme cold is also concerning due to climate change.

However, there are some limitations in this study. First, because of the limitation of geographical information, exposure of temperature was assessed at the city level, which may induce some degree of exposure misclassification. However, this might not attenuate the risk estimates [40]. Second, since the research objects are middle-aged and older people over 45 years old, the results cannot represent the impact of temperature on depression in younger people. Third, the long-term temperature exposure could be affected by other potential unknown confounding factors, which may lead to inaccurate estimation. 

## 5. Conclusions

In a longitudinal study of an aging population, we observed that long-term residence in regions with extreme cold or heat was associated with the risk of depressive symptoms. The results also indicated that people living in northern China have attenuated risk of low apparent temperature. Middle-aged people and people with lower socioeconomic status are more vulnerable to extreme heat, and older people are more vulnerable to extreme cold. Under climate change and the aging of the population, these findings provided implications for policy making and adaptive strategies. Further studies are needed to investigate the underlying mechanisms for the reported association. 

## Figures and Tables

**Table 1:** Summary statistics of the participants’ baseline characteristics.

Characteristics	Participants Followed with Depressive Symptoms	Healthy Participants	Total Participants at Baseline
**Subjects**	2457	3143	5600
** Individual-level variables**			
Age (mean (SD)), yr	57.55 (8.46)	56.90 (8.04)	57.18 (8.23)
Sex = Male	1080 (44.0%)	1849 (58.8%)	2936 (52.4%)
Marriage status = married	2248 (91.5%)	2943 (93.6%)	5191 (92.7%)
Education attainment = higher than primary school	1377 (56.0%)	2243 (71.4%)	2279 (40.7%)
Urbanicity = urban residence	829 (33.7%)	1394 (44.4%)	2223 (39.7%)
Household income (mean (SD)), CNY	13,111.11 (29006.50)	16,248.56 (27902.13)	14,872.01 (28432.06)
**Area-level variables**			
Geographic location = living in the southern China	1429 (58.2%)	1501 (47.8%)	2930 (52.3%)
City level GDP (mean (SD)), CNY	15.86 (18.53)	20.43 (23.28)	18.43 (21.44)
**Meteorological variables**			
Apparent temperature (mean (SD)), °C	13.06 (6.42)	12.55 (5.98)	12.75 (6.03)
Ambient temperature (mean (SD)), °C	13.91 (5.02)	13.49 (4.73)	137.29 (47.46)
Relative humidity (mean (SD)), %	67.95 (7.86)	66.90 (7.36)	66.49 (7.16)
Sunlight (mean (SD)), hours/day	49.07 (12.16)	50.63 (11.06)	50.92 (10.76)
**Extreme hot indicators**			
Tropical nights (mean (SD)), days	76.87 (53.56)	75.13 (47.87)	76.30 (49.65)
Summer days (mean (SD)), days	135.92 (45.84)	133.27 (40.99)	131.48 (41.57)
Warm nights (mean (SD)), days	32.45 (4.27)	32.07 (4.10)	31.13 (7.53)
Warm days (mean (SD)), days	33.23 (5.67)	31.75 (5.26)	34.89 (9.40)
Warm spell duration (mean (SD)), days	28.60 (6.47)	27.13 (6.20)	30.08 (10.33)
**Extreme cold indicators**			
Frost nights (mean (SD)), days	69.21 (62.03)	77.75 (59.13)	73.92 (58.36)
Ice days (mean (SD)), days	20.78 (35.61)	23.58 (36.60)	20.95 (34.13)
Cool nights (mean (SD)), days	46.83 (4.42)	47.21 (4.10)	48.81 (6.00)
Cool days (mean (SD)), days	54.34 (5.70)	53.98 (5.29)	51.24 (5.56)
Cold spell duration (mean (SD)), days	42.98 (5.13)	43.40 (4.87)	45.35 (6.22)

Note: yr = years; SD = standard difference; GDP = gross domestic product.

**Table 2:** Cox regression models of time to having depressive symptoms, 2011–2018.

Variable	Hazard Ratio (95% CI)
**Apparent temperature**	
High temperature	1.027(1.013–1.041) ***
Low temperature	1.023(1.011–1.035) ***
**Extreme hot**	
Tropical nights	1.344(1.127–1.602) ***
Summer days	0.581(0.265–1.273)
Warm nights	1.048(0.856–1.284)
Warm days	0.801(0.63–1.017) *
Warm spell durations	0.836(0.698–1.002) *
**Extreme cold**	
Frost nights	1.166(0.981–1.386) *
Ice days	1.289(1.114–1.491) ***
Cool nights	2.064(1.507–2.825) ***
Cool days	1.315(1.061–1.631) **
Cold spell durations	1.645(1.306–2.072) ***

Note: *, **, and *** indicate significance at 10%, 5%, and 1% levels, respectively.

**Table 3:** Hazards ratios of depressive symptoms, each unit increase in low temperature and annual change in cool nights by baseline characteristics.

Characteristics	Subgroup	High Temperature		Low Temperature	
		Hazard Ratio (95% CI)	*p*-Interaction	Hazard Ratio (95% CI)	*p*-Interaction
Sex	Female	1.018(1.001,1.037)	Ref.	1.028(1.013,1.044)	Ref.
	Male	1.036(1.017,1.055)	0.146	1.016(0.999,1.032)	0.218
Age	<65 yr	1.024(1.008,1.039)	Ref.	1.021(1.008,1.034)	Ref.
	≥65 yr	1.037(1.01,1.064)	0.38	1.031(1.005,1.057)	0.491
Urbanicity of residence	Rural	1.022(1.005,1.038)	Ref.	1.023(1.009,1.037)	Ref.
	Urban	1.037(1.015,1.059)	0.24	1.022(1.004,1.041)	0.911
Household income	Above average	1.021(0.999,1.044)	Ref.	1.012(0.992,1.033)	Ref.
	Below average	1.029(1.013,1.046)	0.557	1.027(1.013,1.041)	0.197
Education attainment	Primary school or below	1.016(0.996,1.036)	Ref.	1.029(1.011,1.046)	Ref.
	Junior school or above	1.035(1.018,1.053)	0.118	1.019(1.005,1.033)	0.351
Geographic location	Living in north	0.862(0.703,1.058)	Ref.	1.031(1.018,1.044)	Ref.
	Living in south	1.011(0.994,1.028)	0.118	1.115(1.053,1.181)	0.014

## Data Availability

The population data (CHARLS) that support the findings of this study are available from (http://charls.pku.edu.cn/index/en.html, accessed 15 March 2021). The climate data that support the findings of this study are available from (http://data.cma.cn/data/cdcdetail/dataCode/SURF_CLI_CHN_MUL_DAY.html, accessed 15 March 2021).

## Supplementary Materials

The following supporting information can be downloaded at: https://www.mdpi.com/article/10.3390/ijerph20043229/s1, Appendix method; Figure S1: Flowchart of the study samples; Figure S2: Cross-validation for the monthly estimates of apparent temperature (2011-2018) in China; Figure S3: Conceptual causal pathway is shown as directed acyclic graph (DAG); Figure S4: Hazards ratios of depressive symptoms each percent increase of annual change of ice days by baseline characteristics; Figure S5: Hazards ratios of depressive symptoms each percent increase of annual change of cool nights by base-line characteristics; Figure S6: Hazards ratios of depressive symptoms each percent increase of annual change of cool days by baseline characteristics; Figure S7: Hazards ratios of depressive symptoms each percent increase of annual change of cool duration by baseline characteristics; Figure S8: The exposure-response curve of long-term ambient temperature exposure and depressive symptoms; Table S1: Definition of extreme temperature indicators; Table S2: Index table of the variables; Table S3: Summary statistics on population characteristics (N = 17,596); Table S4: The associations between temperature and depressive symptoms in sensitive analysis, by using 12 scores as a cut-off of depressive symptoms, or 2-year moving average apparent temperature before survey time.
