# Supplementary material for: Long-Term Apparent Temperature, Extreme Temperature Exposure, and Depressive Symptoms: A Longitudinal Study in China
Source: Int J Environ Res Public Health. 2023 Feb 12;20(4):3229. doi: 10.3390/ijerph20043229 (PMC9962105; doi:10.3390/ijerph20043229)
Supplement: Supplementary file 1 [file ijerph-20-03229-s001.zip › ijerph-2161321-supplementary.pdf]

International Journal of
Environmental Research and Public Health

MDPI

Supplemental Materials:

Long-term apparent temperature, extreme temperature exposure and depressive symptoms: A longitudinal study in China

Jianbo Jin, Zhihu Xu, Ru Cao, Yuxin Wang, Qiang Zeng, Xiaochuan Pan, Jing Huang, Guoxing Li

Contents

Appendix method

Figure S1. Flowchart of the study samples.

Figure S2. Cross-validation for the monthly estimates of apparent temperature (2011-2018) in China

Figure S3. Conceptual causal pathway is shown as directed acyclic graph (DAG)

Figure S4. Hazards ratios of depressive symptoms each percent increase of annual change of ice days by baseline characteristics

Figure S5. Hazards ratios of depressive symptoms each percent increase of annual change of cool nights by baseline characteristics

Figure S6. Hazards ratios of depressive symptoms each percent increase of annual change of cool days by baseline characteristics

Figure S7. Hazards ratios of depressive symptoms each percent increase of annual change of cool duration by baseline characteristics

Figure S8. The exposure-response curve of long-term ambient temperature exposure and depressive symptoms.

Table S1. Definition of extreme temperature indicators.

Table S2. Index table of the variables.

Table S3. Summary statistics on population characteristics (N=17,596).

Table S4. The associations between temperature and depressive symptoms in sensitive analysis, by using 12 scores as a cut-off of depressive symptoms, or 2-year moving average apparent temperature before survey time.

Appendix method

The Center for Epidemiologic Studies Depression Scale (CES-D10)

Respondents were asked how frequently in the last week they: were bothered by things; had trouble concentrating on things; felt depressed; felt everything was an effort; felt hopeful about future; felt fearful; had restless sleep; was happy; felt lonely; and could not get going. After reverse scoring the two positively worded items, we assigned a score from 0 to 3 for each item as follows: 0 for 0 days, 1 for 1–2 days; 2 for 3–4 days; 3 for 5–7 days. Scores of these 10 items were summed to create an additive scale score ranging from 0 to 30, with higher scores indicating more depressive symptoms.

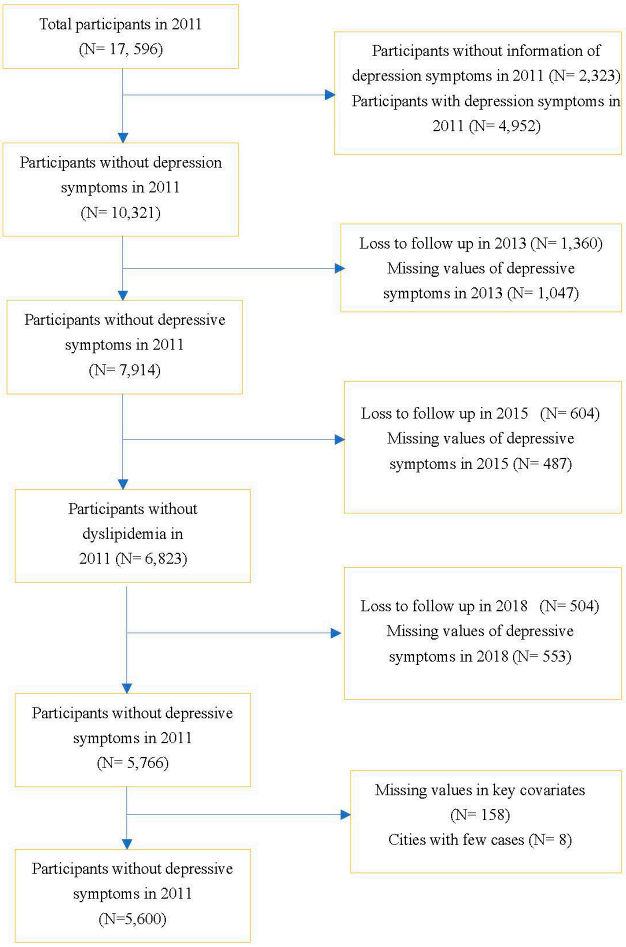

Figure S1. Flowchart of the study samples.

Two cities, Shenzhen (subjects:4; incidence: 0.20) and Lanzhou (subjects: 4; incidence :0.33), were excluded for potential impact on the distribution of samples (average number of subjects: 45.16; average incidence: 0.03)

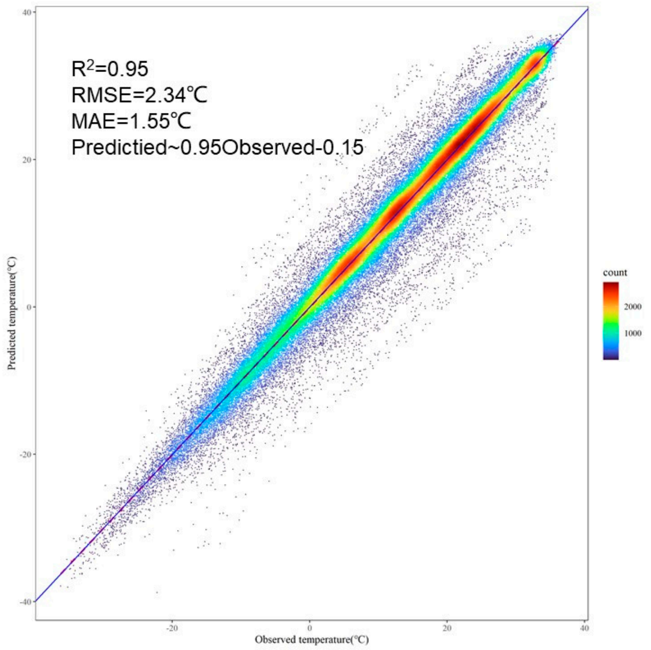

Figure S2. Cross-validation for the monthly estimates of apparent temperature (2011-2018) in China.

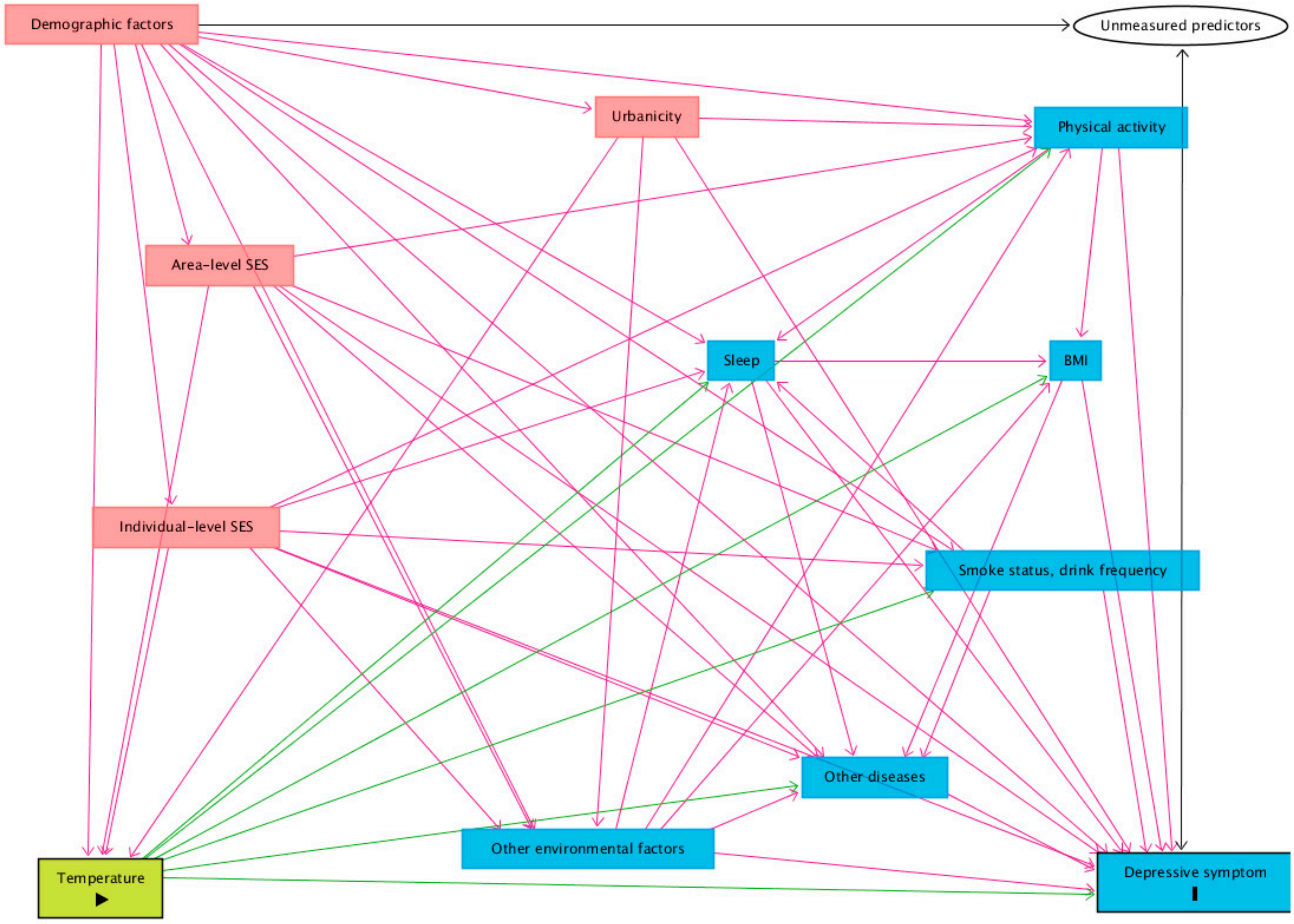

Figure S3. Conceptual causal pathway is shown as directed acyclic graph (DAG).

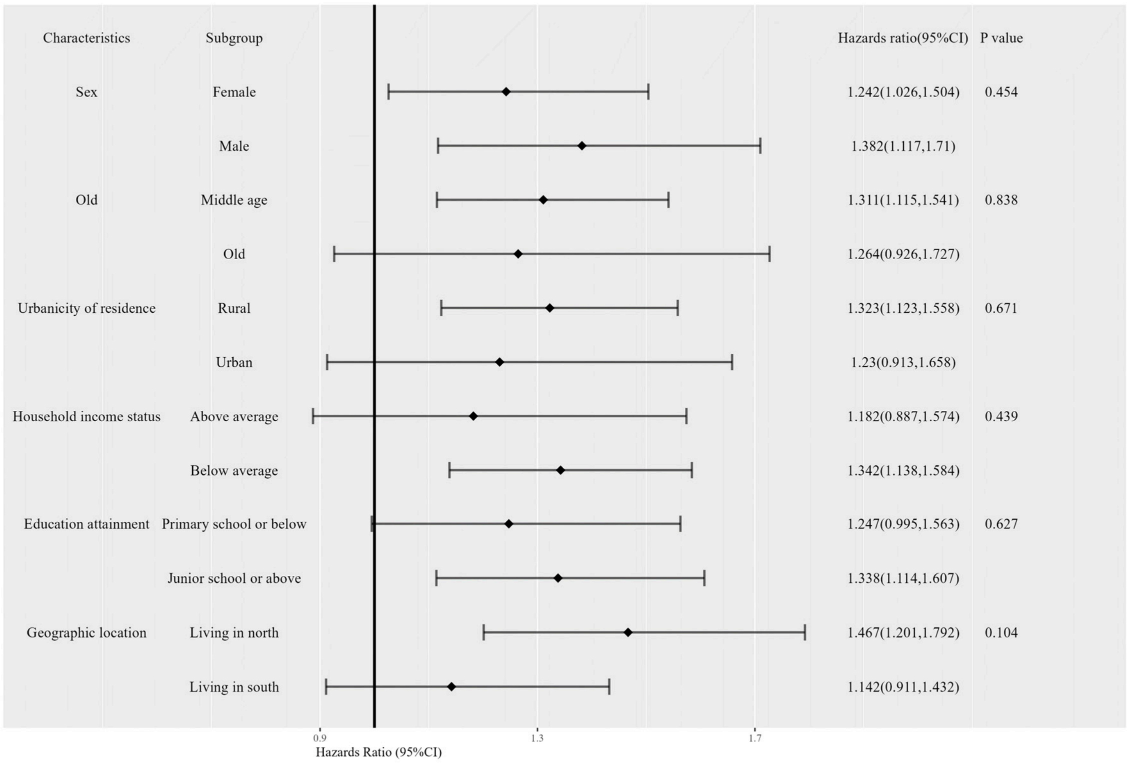

Figure S4. Hazards ratios of depressive symptoms each percent increase of annual change of ice days by baseline characteristics

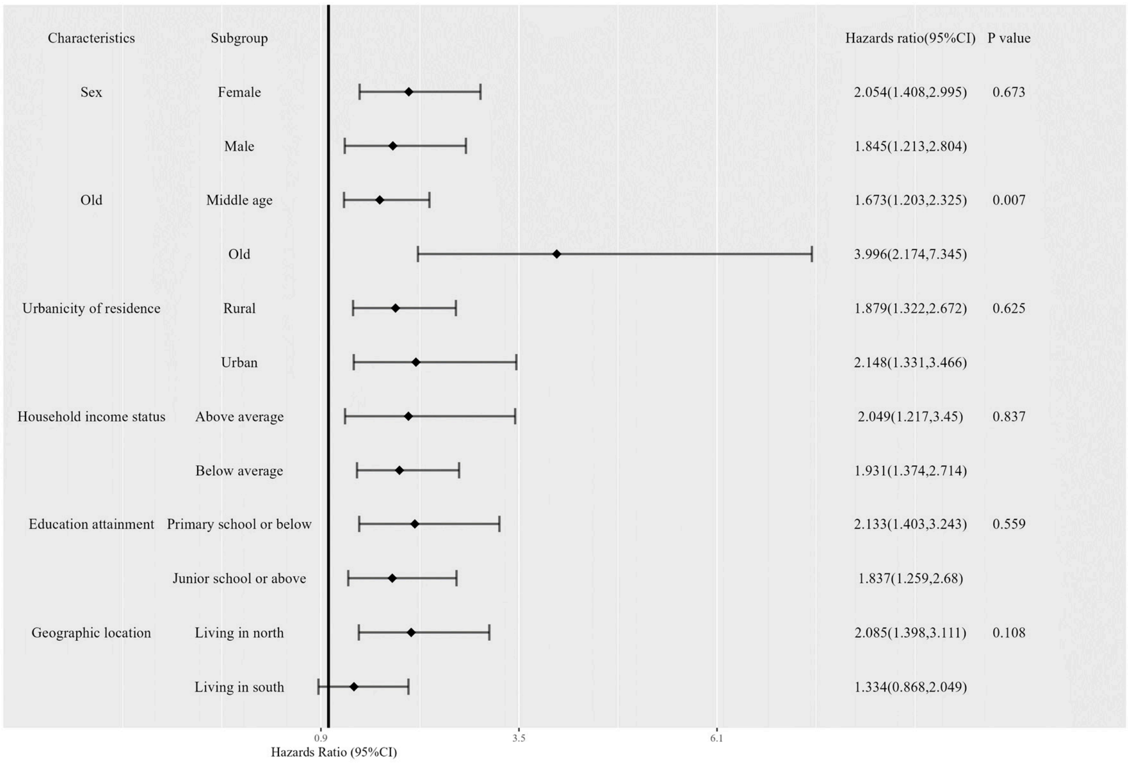

Figure S5. Hazards ratios of depressive symptoms each percent increase of annual change of cool nights by baseline characteristics

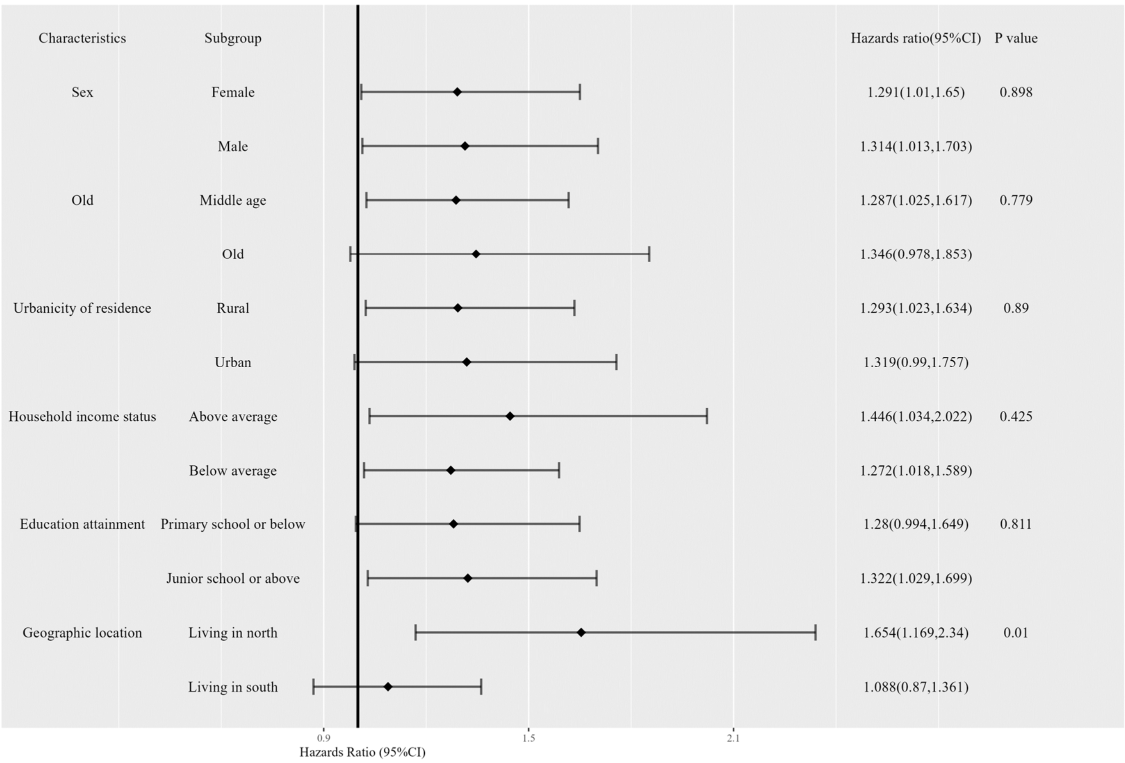

Figure S6. Hazards ratios of depressive symptoms each percent increase of annual change of cool days by baseline characteristics

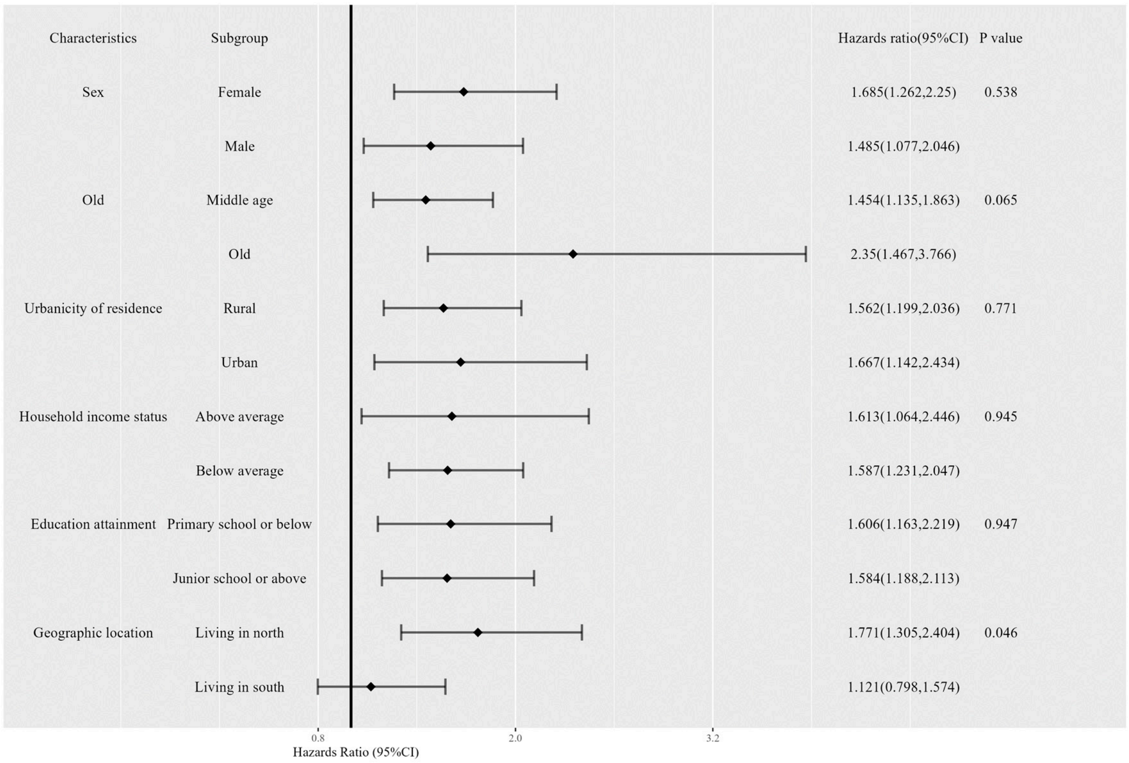

Figure S7. Hazards ratios of depressive symptoms each percent increase of annual change of cool duration by baseline characteristics

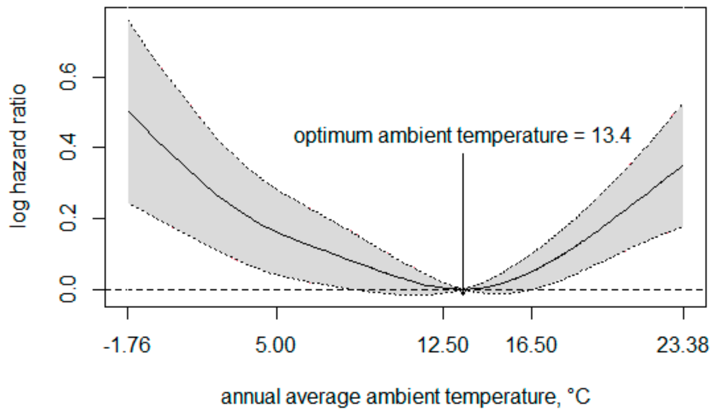

Figure S8. The exposure-response curve of long-term ambient temperature exposure and depressive symptoms. Age, sex, marriage status, household income, education attainment, urbanicity of residence, gross domestic product (GDP), and annual average sunlight hours were adjusted. The solid line represents log hazard ratio, and gray zone indicates 95% confidence interval.

Table S1. Definition of extreme temperature indicators

Name	Definition	Unit
**Extreme hot indicators**		
Tropical nights	Daily minimum temperature > 20 °C	Days
Summer days	Daily maximum temperature > 25 °C	Days
Warm nights	Daily minimum temperature > 90th percentile	Days
Warm days	Daily maximum temperature > 90th percentile	Days
Warm spell duration	Daily maximum temperature > 90th percentile for ≥ 2 consecutive days	Days
**Extreme cold indicators**		
Frost nights	Daily minimum temperature < 0 °C	Days
Ice days	Daily maximum temperature < 0 °C	Days
Cool nights	Daily minimum temperature < 10th percentile	Days
Cool days	Daily maximum temperature < 10th percentile	Days
Cold spell duration	Daily minimum temperature < 10th percentile for ≥ 2 consecutive days	Days

Notes: 10th/90th percentile was calculated as the calendar day 10th/90th percentile of the daily temperature distribution in the period of 2010–2018 for each city

Table S2. Index table of the variables.

Variable name	Definition
**Individual-level variables**	
Age	Unit: years
gender	Female = 0, male = 1
Marriage status	Single = 0, married = 1
Education attainment	Primary school or lower = 0, Higher than primary school = 1
Urbanicity	Rural residence = 0, Urban residence = 1
Household income	Unit: yuan
**Area-level variables**	
Geographic location	Living in the northern China = 0, Living in the southern China = 1
City level GDP	Unit: million yuan
**Meteorological variables**	
Apparent temperature	Unit: ℃
Ambient temperature	Unit: ℃
Relative humidity	Unit: %
Sunlight	Unit: hours/day
**Extreme hot indicators**	
Tropical nights	Unit: days
Summer days	Unit: days
Warm nights	Unit: days
Warm days	Unit: days
Warm spell duration	Unit: days
**Extreme cold indicators**	
Frost nights	Unit: days
Ice days	Unit: days
Cool nights	Unit: days

Cool days	Unit: days
Cold spell duration	Unit: days

Abbreviation: GDP = gross domestic product.

Table S3. Summary statistics on population characteristics (N=17,596)

Characteristics	Included	Excluded	Total
**Subjects**	5600	11996	17596
**Individual-level variables**			
Age (mean (SD)), yr	57.18 (8.23)	59.92 (10.82)	59.05 (10.15)
Sex = Male	2936 (52.4%)	5493 (45.8%)	8429 (47.9%)
Marriage status = Married	5191 (92.7%)	10199 (85.1%)	15390 (87.5%)
Education attainment = Higher than primary school	2279 (40.7%)	3595 (30.0%)	5874 (33.4%)
Urbanicity = Urban residence	2223 (39.7%)	4874 (40.6%)	7097 (40.3%)
Household income (mean (SD)), yuan	14872.01 (28432.06)	14826.25 (33401.62)	14840.96 (31888.18)
**Area-level variables**			
Geographic location = Living in the southern China	2930 (52.3%)	7123 (59.4%)	10053 (57.1%)
City level GDP (mean (SD)), million yuan	18.43 (21.44)	18.74 (24.12)	18.64 (23.30)
**Meteorological variables**			
Apparent temperature (mean (SD)), ℃	12.75 (6.03)	13.22 (6.35)	13.07 (6.25)
Ambient temperature (mean (SD)), ℃	137.29 (47.46)	140.82 (49.65)	139.69 (48.99)
Relative humidity (mean (SD)), %	66.49 (7.16)	67.51 (7.28)	67.19 (7.26)
Sunlight (mean (SD)), hours/day	50.92 (10.76)	49.85 (11.10)	50.19 (11.00)
**Extreme hot indicators**			
Tropical nights (mean (SD)), days	76.30 (49.65)	80.20 (53.11)	78.96 (52.06)
Summer days (mean (SD)), days	131.48 (41.57)	134.74 (43.98)	133.70 (43.25)
Warm nights (mean (SD)), days	31.13 (7.53)	31.47 (7.39)	31.36 (7.44)
Warm days (mean (SD)), days	34.89 (9.40)	36.42 (9.85)	35.93 (9.74)
Warm spell duration (mean (SD)), days	30.08 (10.33)	31.86 (10.68)	31.29 (10.61)
**Extreme cold indicators**			
Frost nights (mean (SD)), days	73.92 (58.36)	68.37 (60.42)	70.14 (59.82)

Ice days (mean (SD)), days	20.95 (34.13)	19.79 (34.25)	20.16 (34.21)
Cool nights (mean (SD)), days	48.81 (6.00)	49.23 (6.02)	49.10 (6.01)
Cool days (mean (SD)), days	51.24 (5.56)	51.76 (5.72)	51.59 (5.68)
Cold spell duration (mean (SD)), days	45.35 (6.22)	45.72 (6.32)	45.61 (6.29)

Note: yr = years; SD = standard difference; GDP = gross domestic product.

Table S4. The associations between temperature and depressive symptoms in sensitive analysis, by using 12 scores as a cut-off of depressive symptoms, or 2-year moving average apparent temperature before survey time.

	Sensitive analysis [a]		Sensitive analysis [b]	
Variable	Hazard Ratio (95% CI)	*P* value	Hazard Ratio (95% CI)	*P* value
**Apparent temperature**				
High temperature	1.019(1.001-1.037)	0.04	1.026(1.012-1.04)	<0.001
Low temperature	1.028(1.016-1.04)	<0.001	1.023(1.011-1.035)	<0.001
**Extreme hot**				
Tropical nights	1.259(1.085-1.461)	0.002	1.25(1.077-1.451)	0.003
Summer days	0.506(0.235-1.087)	0.081	0.633(0.31-1.295)	0.211
Warm nights	0.932(0.797-1.091)	0.38	1.006(0.868-1.164)	0.941
Warm days	0.793(0.636-0.988)	0.039	0.838(0.683-1.029)	0.091
Warm spell duration	0.828(0.695-0.987)	0.035	0.866(0.737-1.017)	0.08
**Extreme cold**				
Frost nights	1.148(0.985-1.338)	0.078	1.161(1.004-1.342)	0.043
Ice days	1.386(1.188-1.617)	<0.001	1.236(1.071-1.427)	0.004
Cool nights	1.97(1.481-2.621)	<0.001	1.924(1.465-2.527)	<0.001
Cool days	1.172(0.951-1.444)	0.136	1.218(1.006-1.476)	0.044
Cold spell duration	1.51(1.255-1.819)	<0.001	1.503(1.253-1.803)	<0.001

Sensitive analysis [a]: Using 12 scores of CEDS-10 as the cut-off of depressive symptoms. Adjusted for model 3 criteria.

Sensitive analysis [b]: Using the 2-year moving average apparent temperature of the year before each survey as the temperature exposure.
